# Supplementary figures and images for: Eps15 Homology Domain Protein 4 (EHD4) is required for Eps15 Homology Domain Protein 1 (EHD1)-mediated endosomal recruitment and fission
Source: PLoS One. 2020 Sep 23;15(9):e0239657. doi: 10.1371/journal.pone.0239657 (PMC7511005; doi:10.1371/journal.pone.0239657)

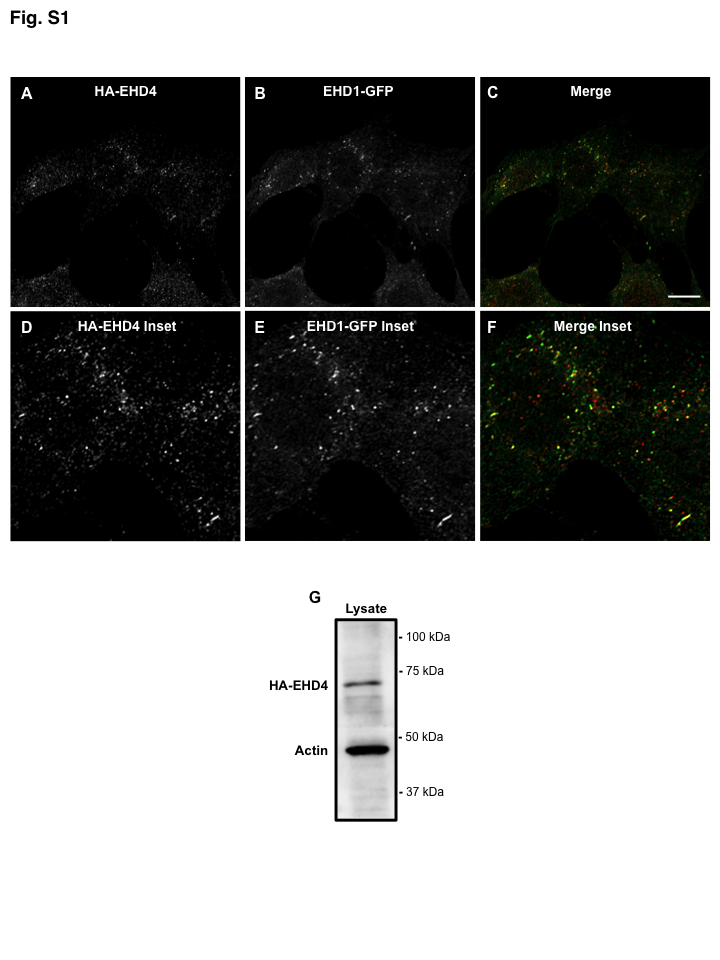

Supplement: S1 Fig — A-F, HA-EHD4 was transfected into CRISPR/Cas9 gene-edited cells expressing endogenous levels of EHD1-GFP on coverslips, fixed and stained with primary antibodies against HA and secondary Alexa-568 antibodies and imaged to detect HA-EHD4 (red; A and inset in D), EHD1-GFP (green; B and inset in E) and then merged to show both channels (C and inset in F). G, Immunoblot shows expression of the correct-sized HA-EHD4 band at ~65 kDa. The Pearson’s Coefficients were calculated with the NIH ImageJ plugin JACoP, and averaged to provide a value of 0.677 (~68%) with a standard deviation of 0.048. (TIFF) [file pone.0239657.s001.tiff]

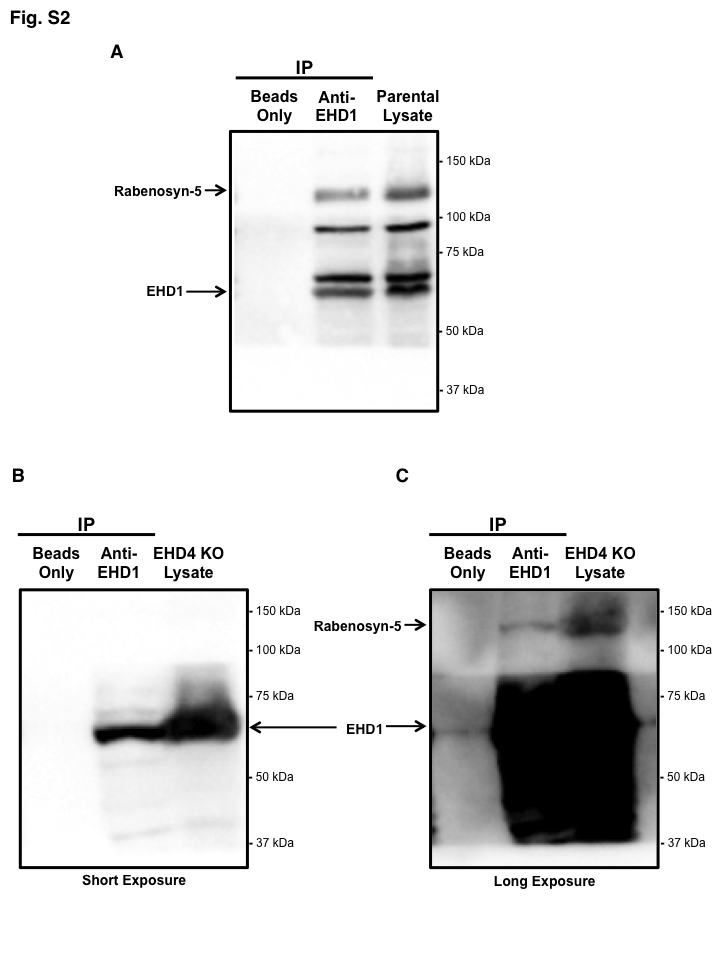

Supplement: S2 Fig — A, NIH3T3 parental cells were grown on a culture dish, pelleted, lysed and either subject directly to SDS PAGE (lysate; right lane), or first immunoprecipitated with beads only (control; left lane) or with anti-EHD1 coupled beads (middle lane) before immunoblotting with anti-Rabenosyn-5 and anti-EHD1. B and C, CRISPR/Cas9 gene-edited NIH3T3 cells knocked out for EHD4 were grown on a culture dish, pelleted, lysed and either subject directly to SDS PAGE (lysate; right lane), or first immunoprecipitated with beads only (control; left lane) or with anti-EHD1 coupled beads (middle lane) before immunoblotting with anti-Rabenosyn-5 and anti-EHD1. C is a darker exposure of the immunoblot depicted in B. (TIFF) [file pone.0239657.s002.tiff]
