# Supplementary material for: Eps15 Homology Domain Protein 4 (EHD4) is required for Eps15 Homology Domain Protein 1 (EHD1)-mediated endosomal recruitment and fission
Source: PLoS One. 2020 Sep 23;15(9):e0239657. doi: 10.1371/journal.pone.0239657 (PMC7511005; doi:10.1371/journal.pone.0239657)
Supplement: S3 Fig — (PDF) [file pone.0239657.s003.pdf]

Fig. 1

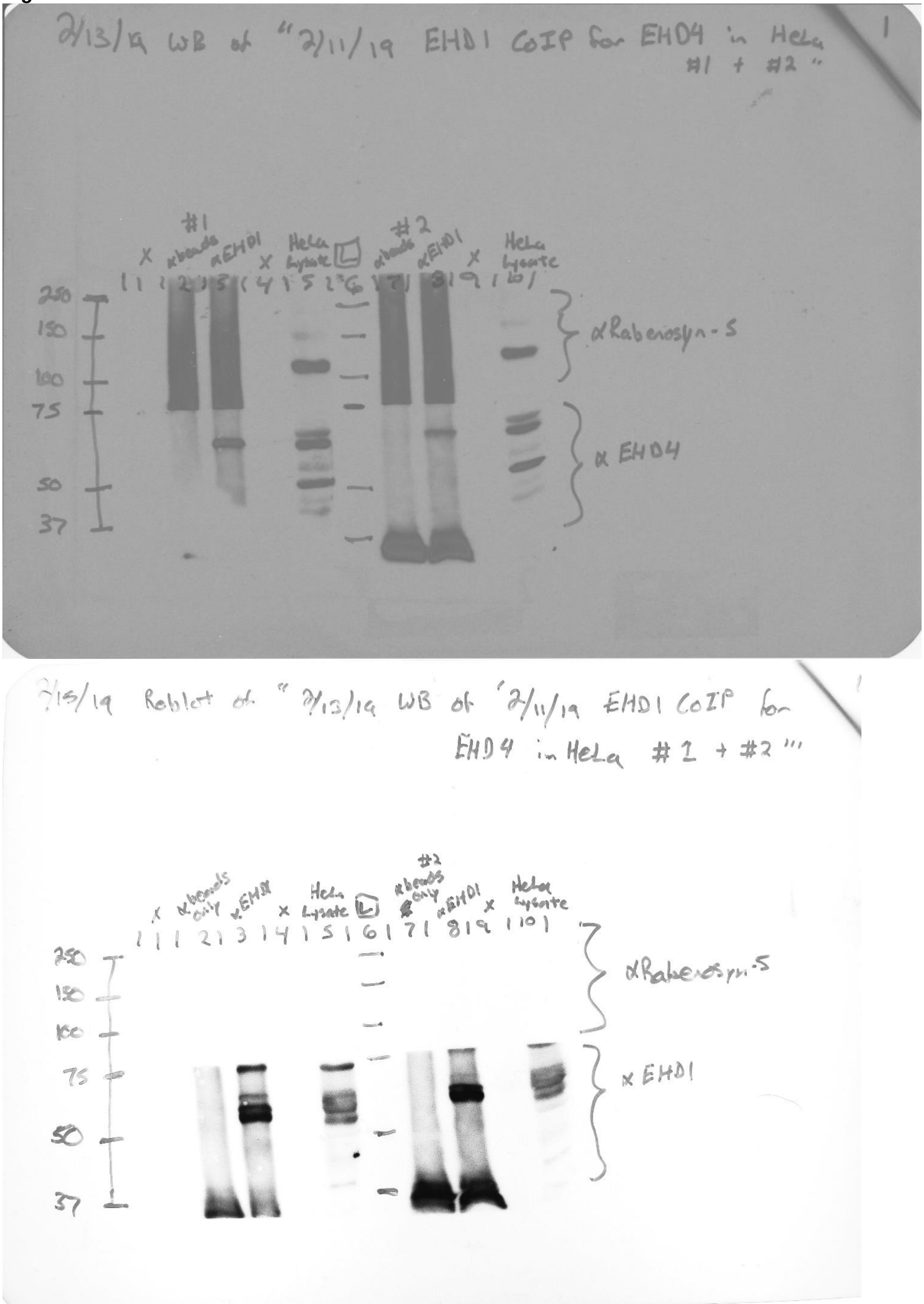
2/13/19 WB of "2/11/19 EHD1 CoIP for EHD4 in HeLa #1 + #2"
#1
#2
HeLa lysate
αRabenosyn-5
α EHD4
2/15/19 Reblot of "2/13/19 WB of '2/11/19 EHD1 CoIP for EHD4 in HeLa #1 + #2'"
α EHD1

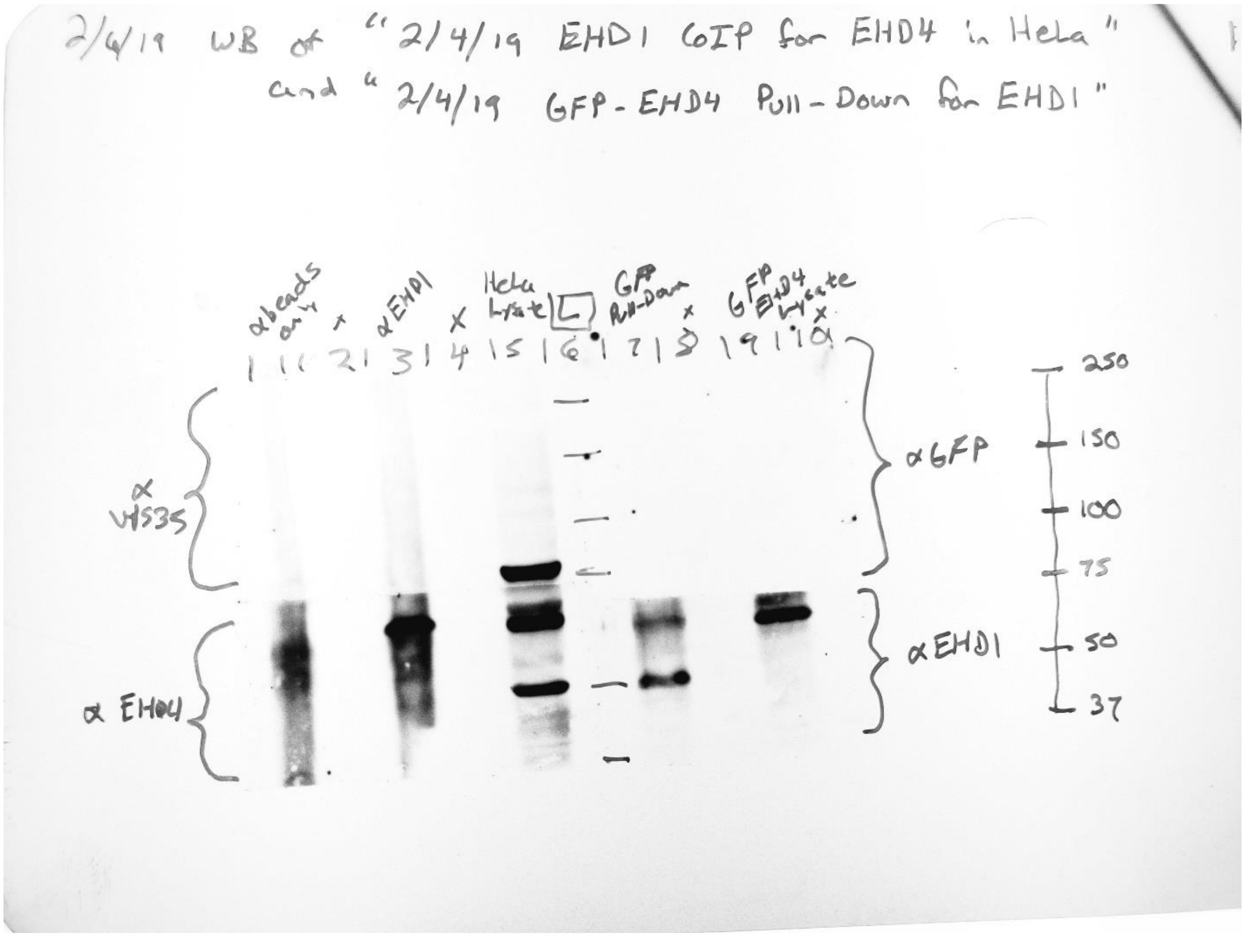
2/4/19 WB of "2/4/19 EHD1 CoIP for EHD4 in HeLa"
and "2/4/19 GFP-EHD4 Pull-Down for EHD1"
α GFP
α EHD1
α VPS35
α EHD4
250
150
100
75
50
37

Fig. 2

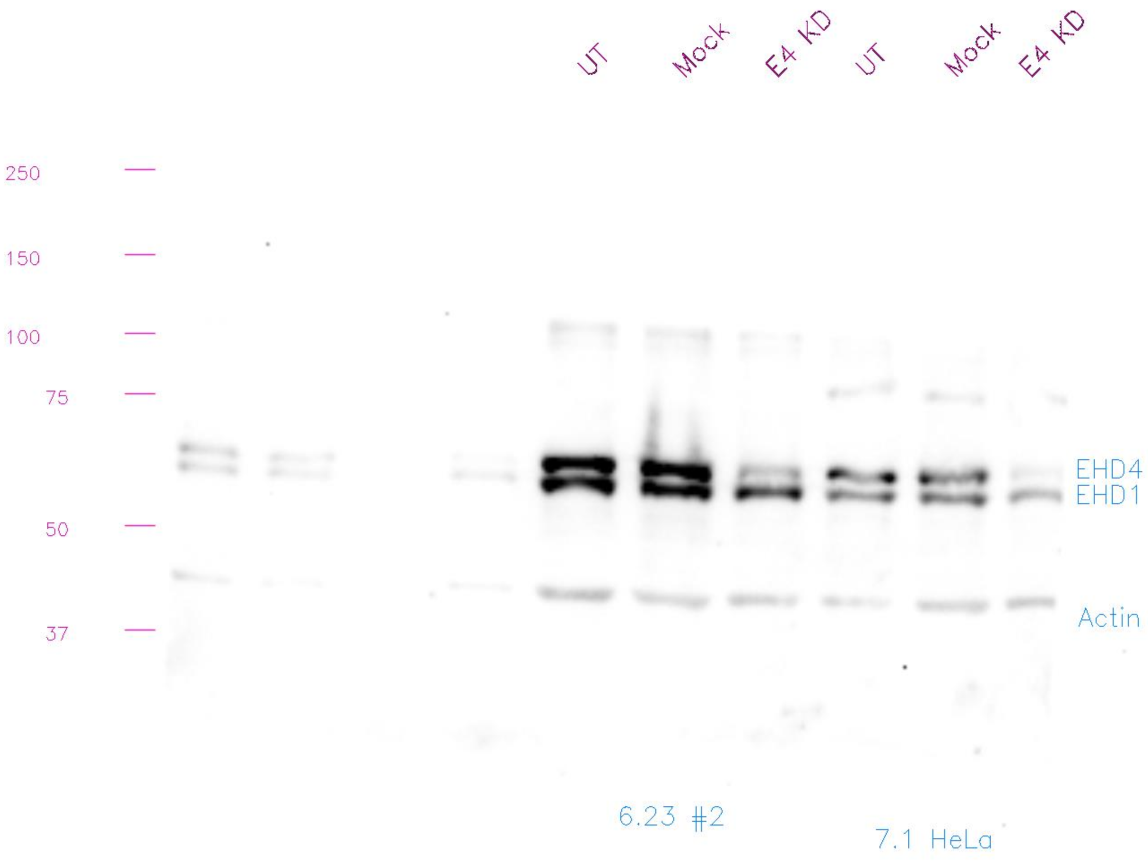
UT
Mock
E4 KD
UT
Mock
E4 KD
250
150
100
75
50
37
EHD4
EHD1
Actin
6.23 #2
7.1 HeLa

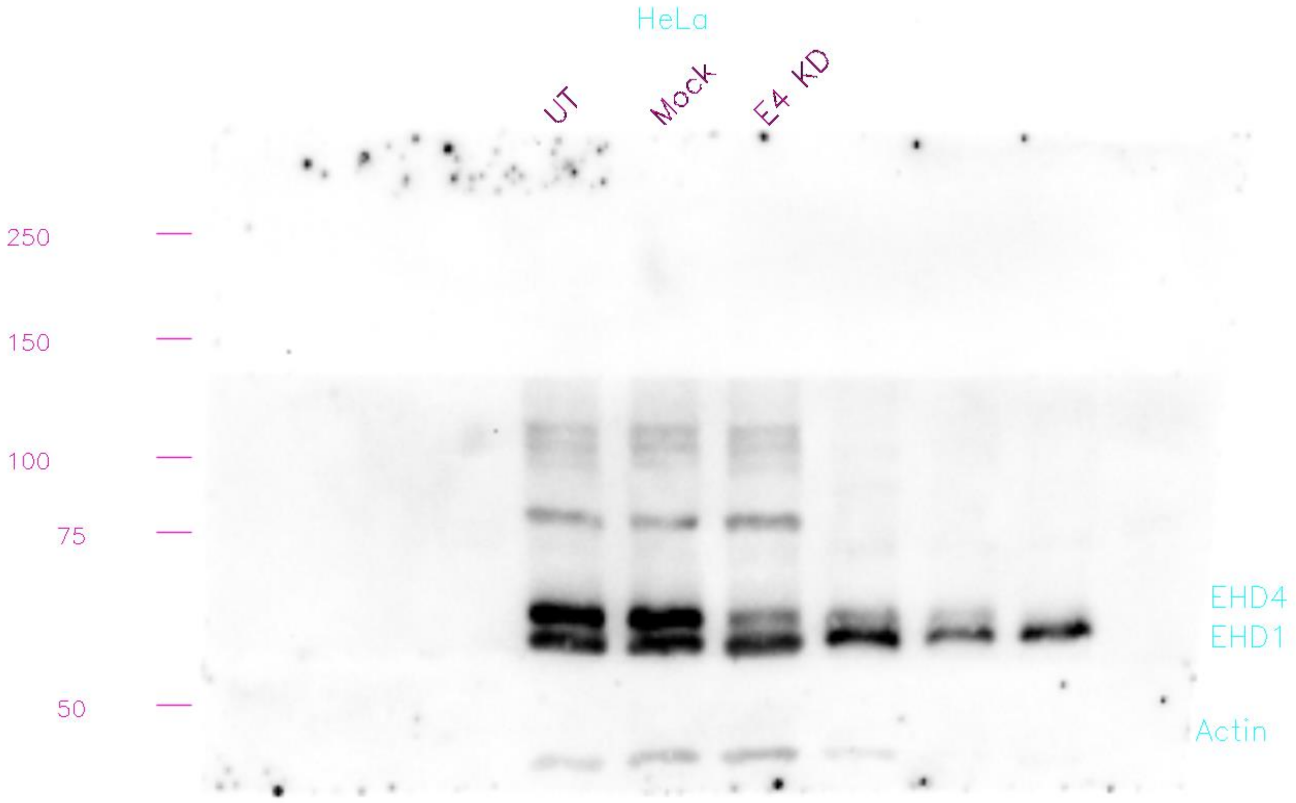
HeLa
UT
Mock
E4 KD
250
150
100
75
50
EHD4
EHD1
Actin

Fig. 3

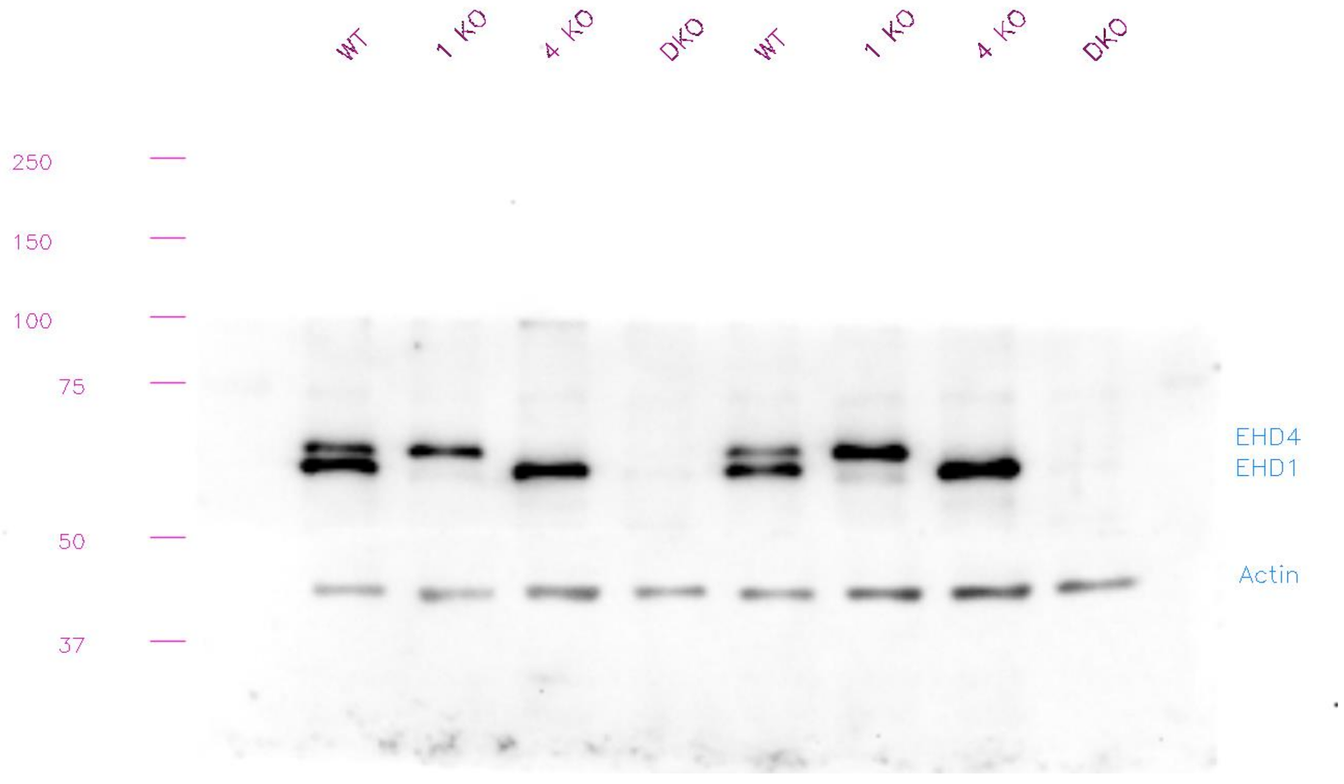
WT
1 KO
4 KO
DKO
WT
1 KO
4 KO
DKO
250
150
100
75
50
37
EHD4
EHD1
Actin

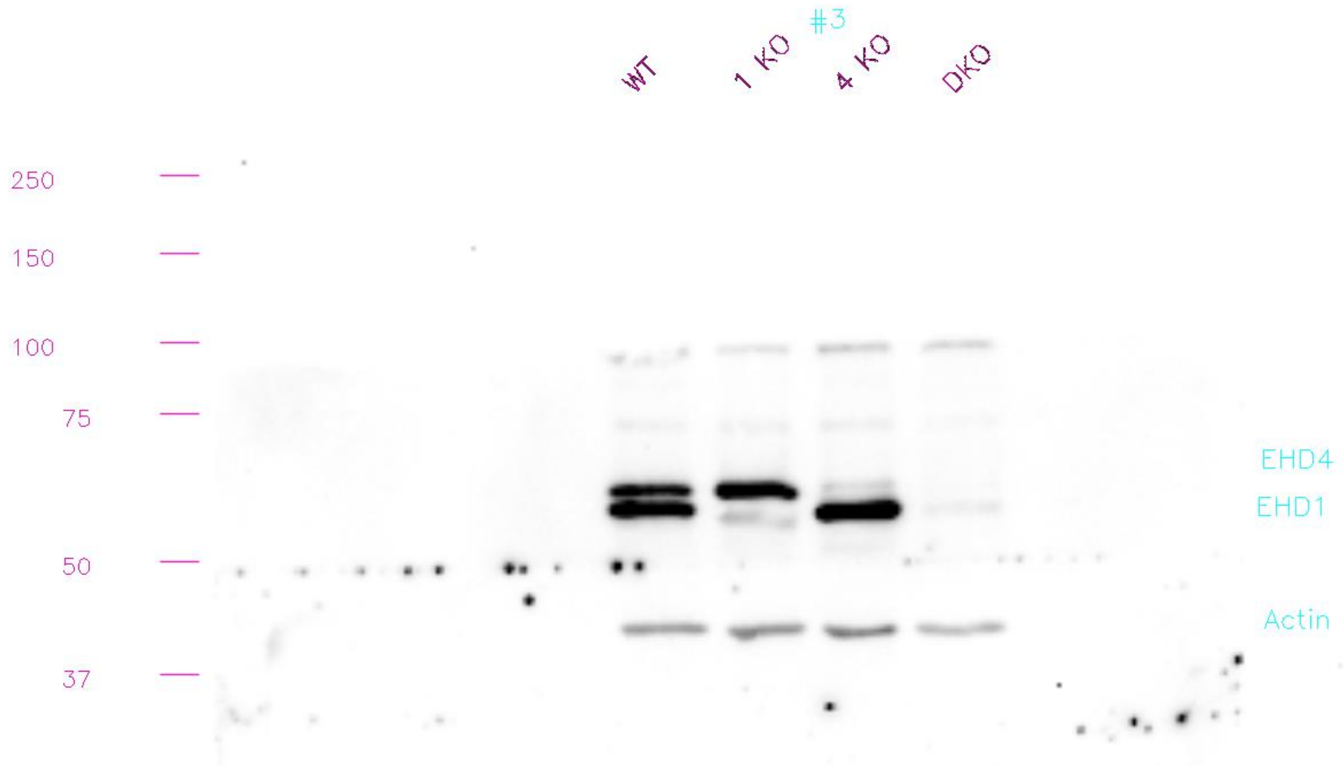
#3
WT
1 KO
4 KO
DKO
250
150
100
75
50
37
EHD4
EHD1
Actin

Fig. 4

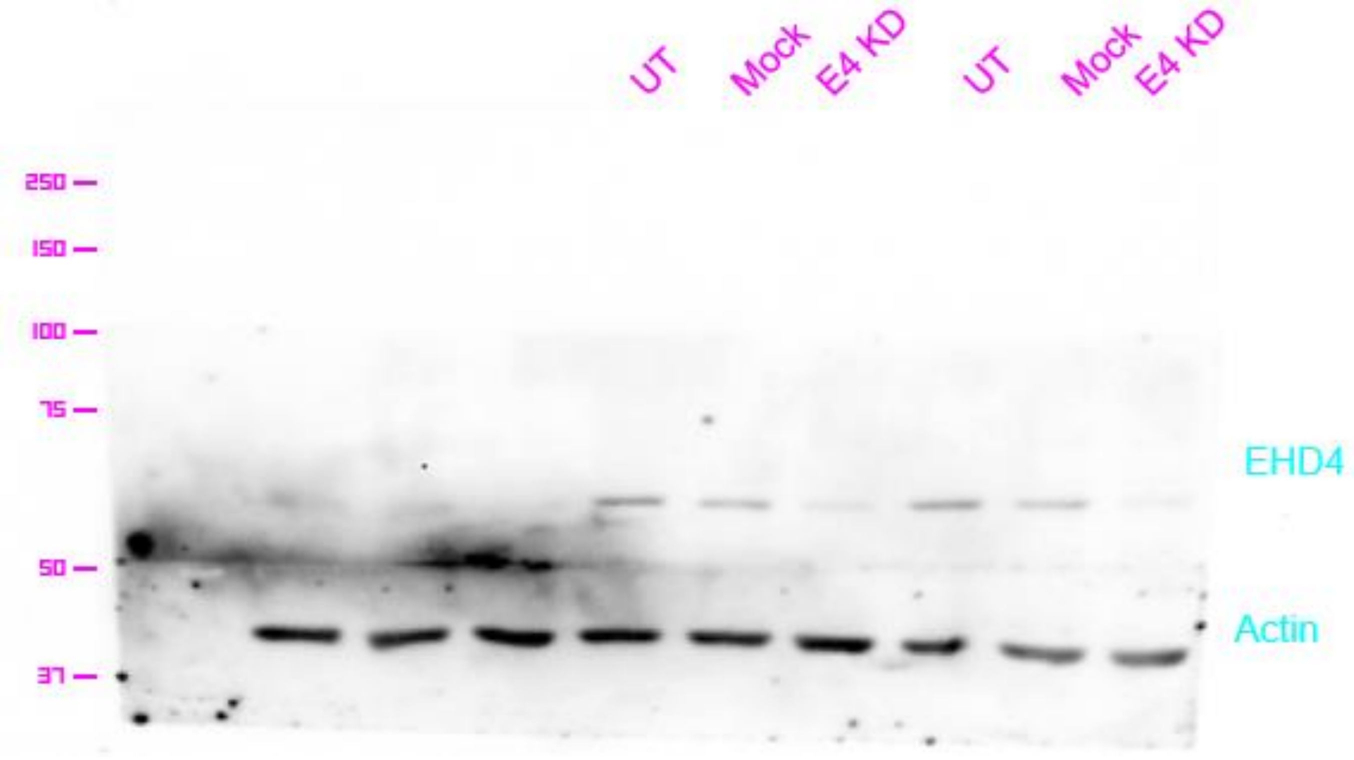
UT
Mock
E4 KD
UT
Mock
E4 KD
250
150
100
75
50
37
EHD4
Actin

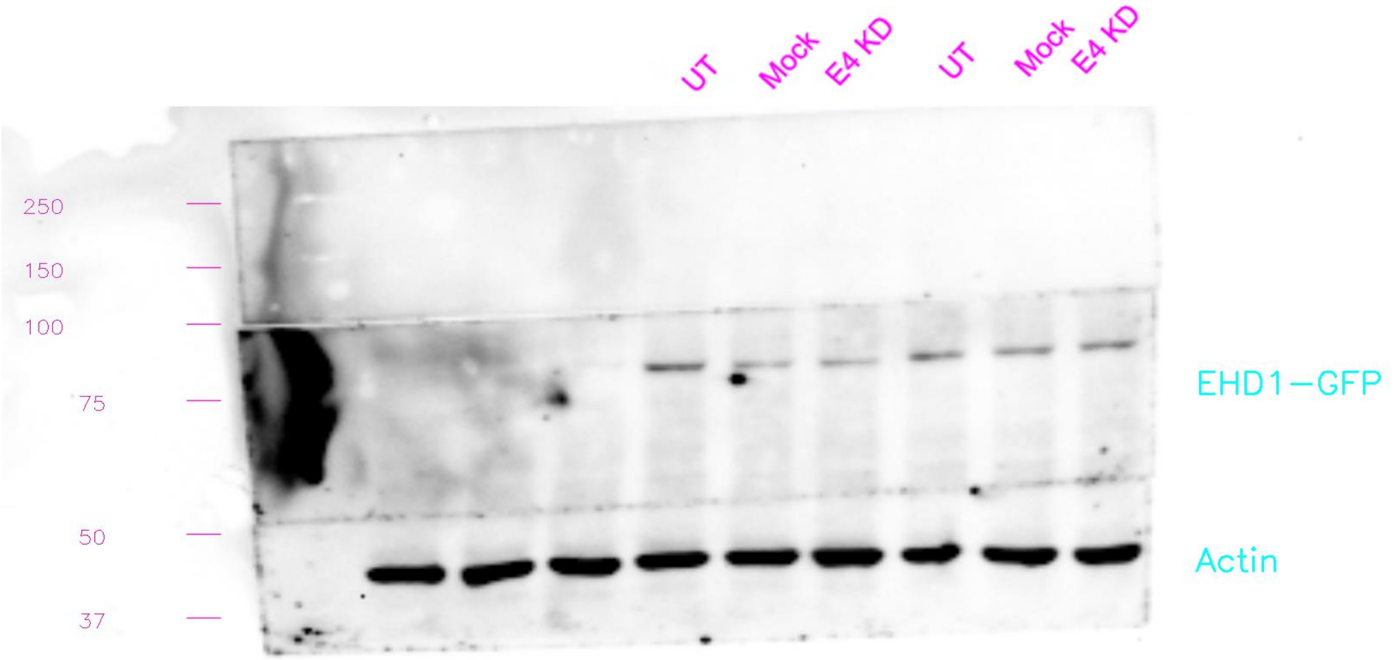
UT
Mock
E4 KD
UT
Mock
E4 KD
250
150
100
75
50
37
EHD1—GFP
Actin

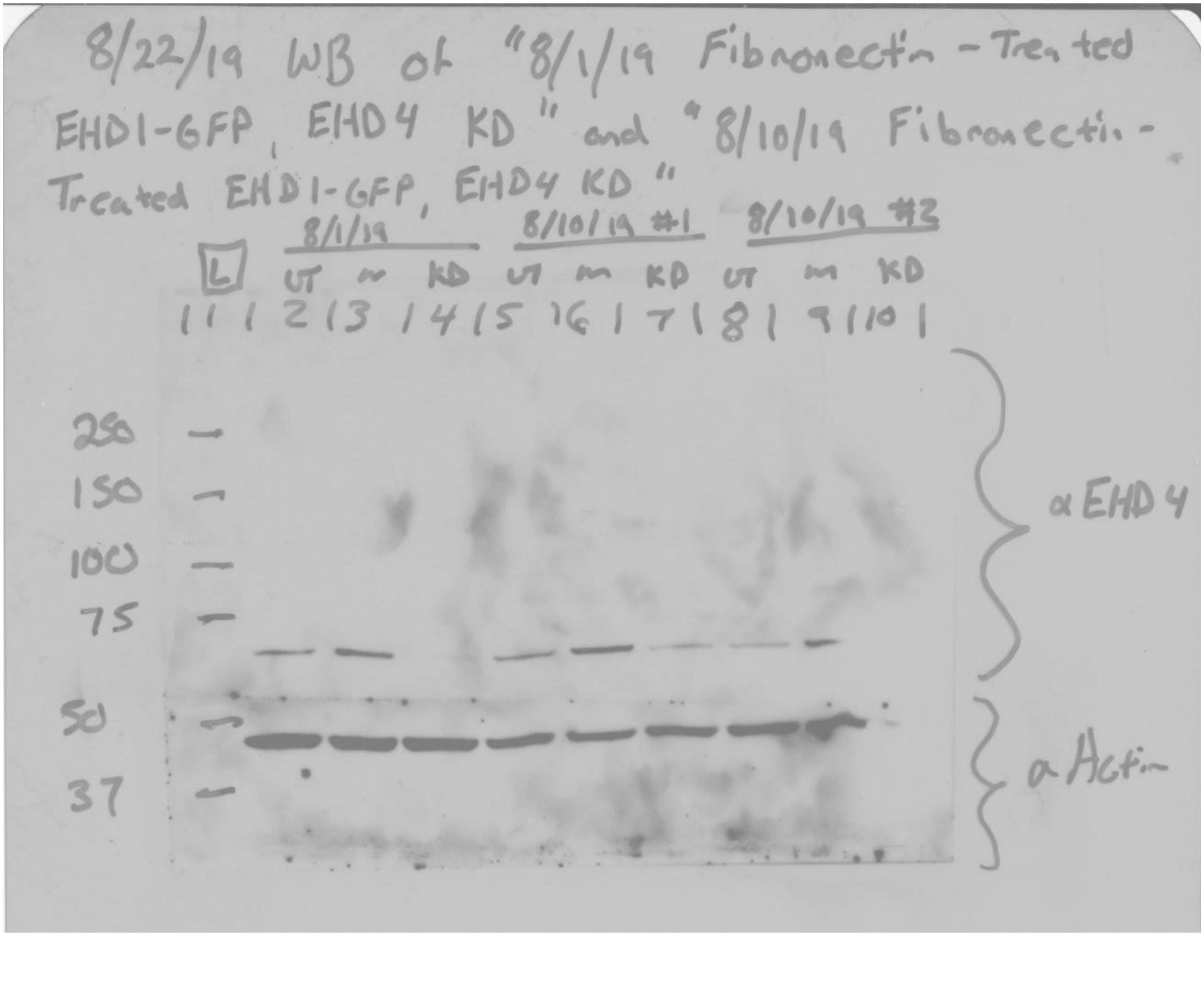
8/22/19 WB of "8/1/19 Fibronectin-Treated EHD1-GFP, EHD4 KD" and "8/10/19 Fibronectin-Treated EHD1-GFP, EHD4 KD"
8/1/19
8/10/19 #1
8/10/19 #2
L
UT m KD UT m KD UT m KD
1 | 2 | 3 | 4 | 5 | 6 | 7 | 8 | 9 | 10
250
150
100
75
50
37
α EHD4
α Actin

Fig. 6

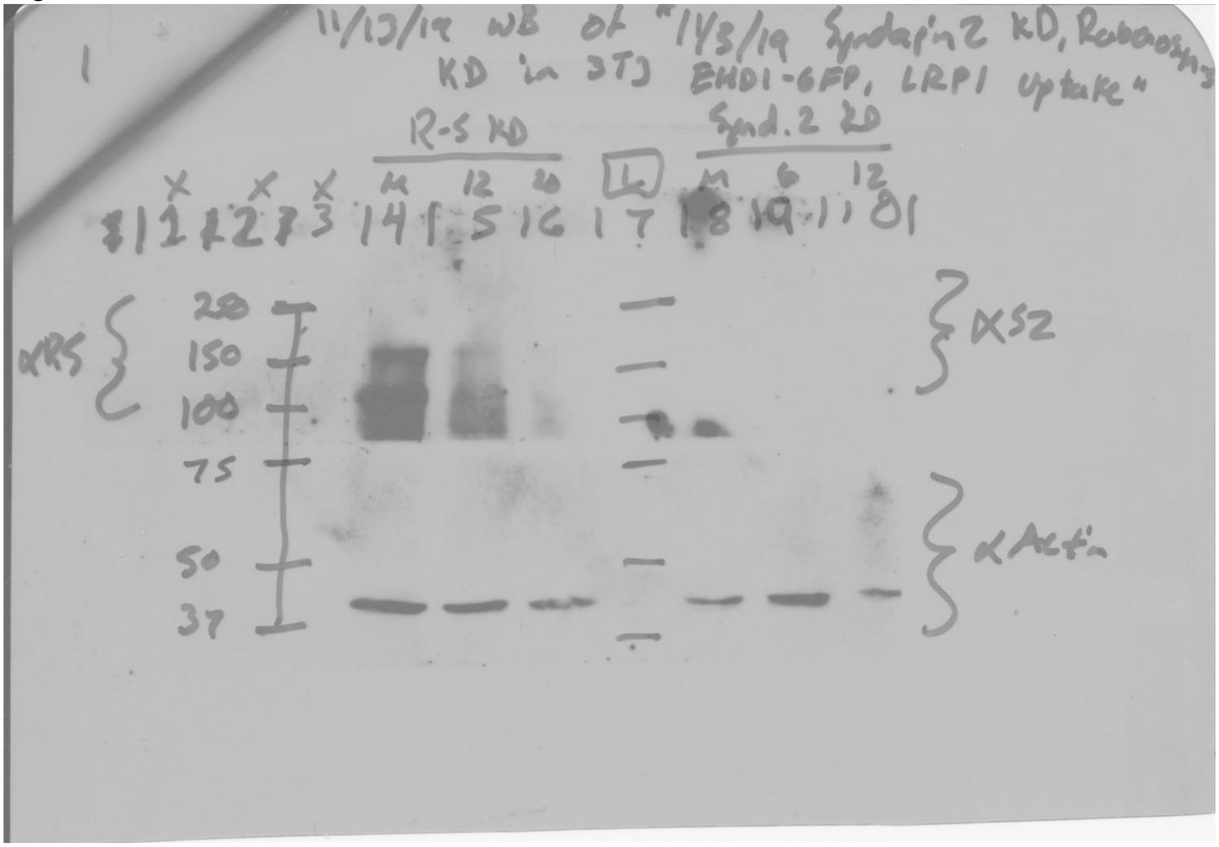

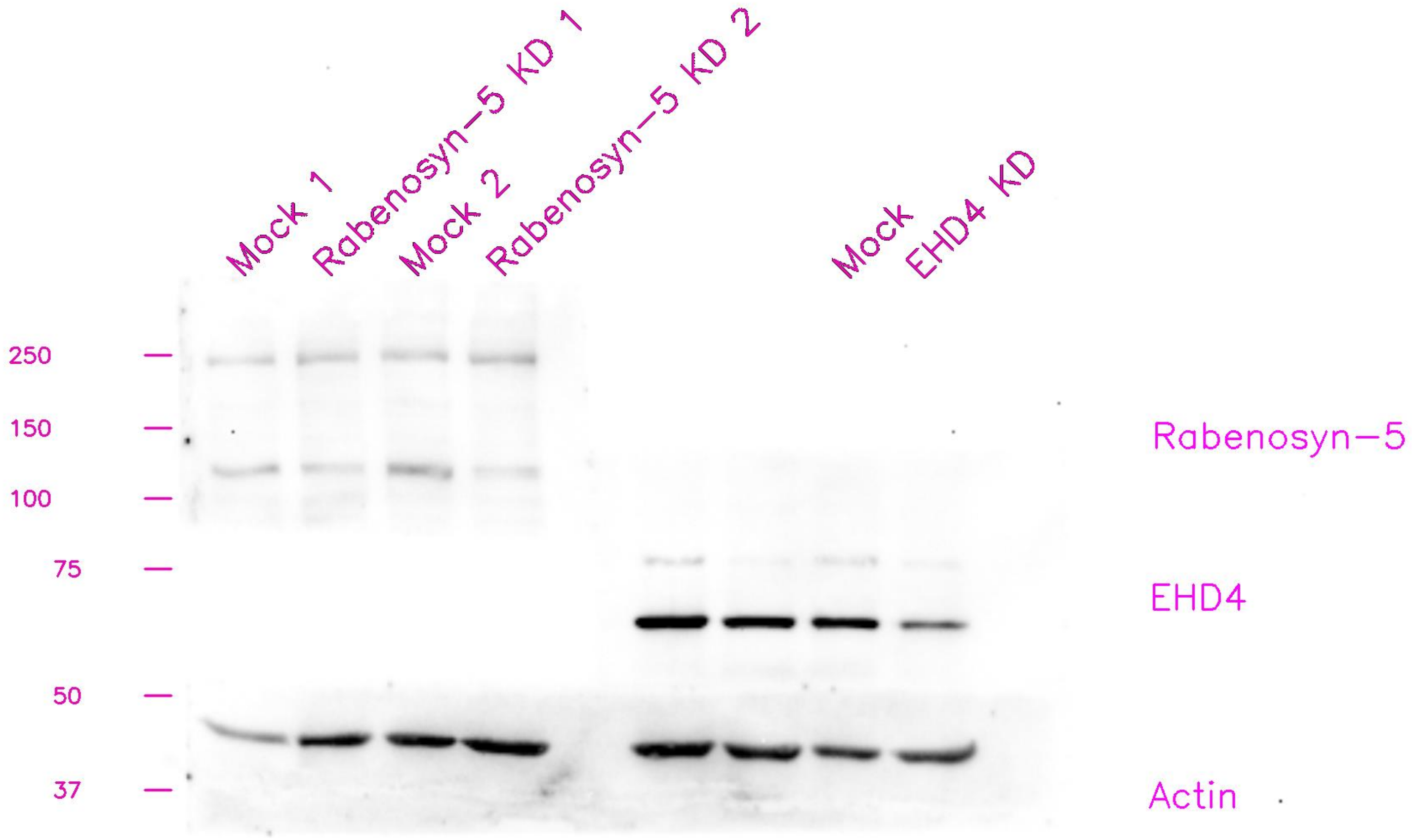

Mock 1
Rabenosyn-5 KD 1
Mock 2
Rabenosyn-5 KD 2
Mock
EHD4 KD
250
150
100
75
50
37
Rabenosyn-5
EHD4
Actin

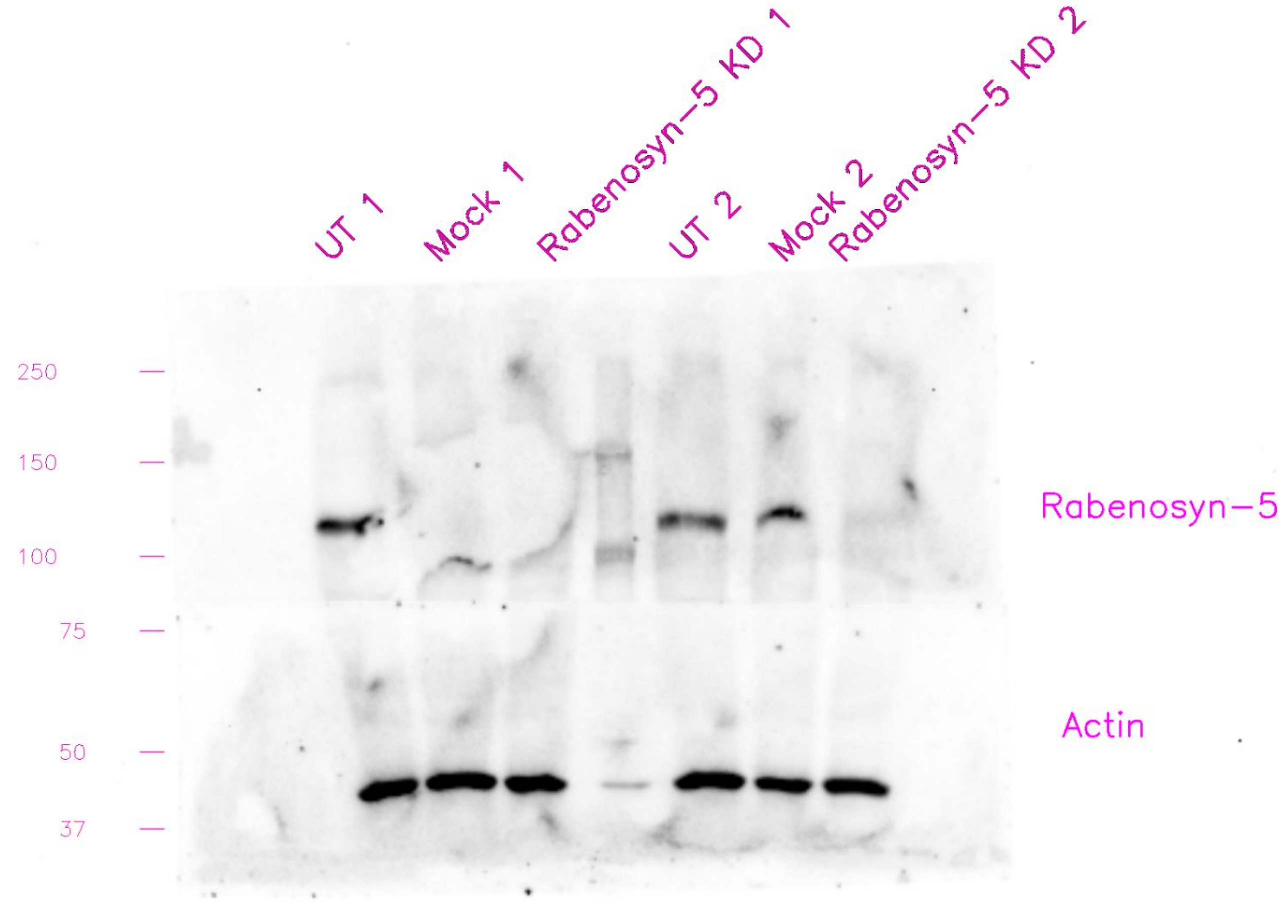
UT 1
Mock 1
Rabenosyn-5 KD 1
UT 2
Mock 2
Rabenosyn-5 KD 2
250
150
100
75
50
37
Rabenosyn-5
Actin

Fig. 7

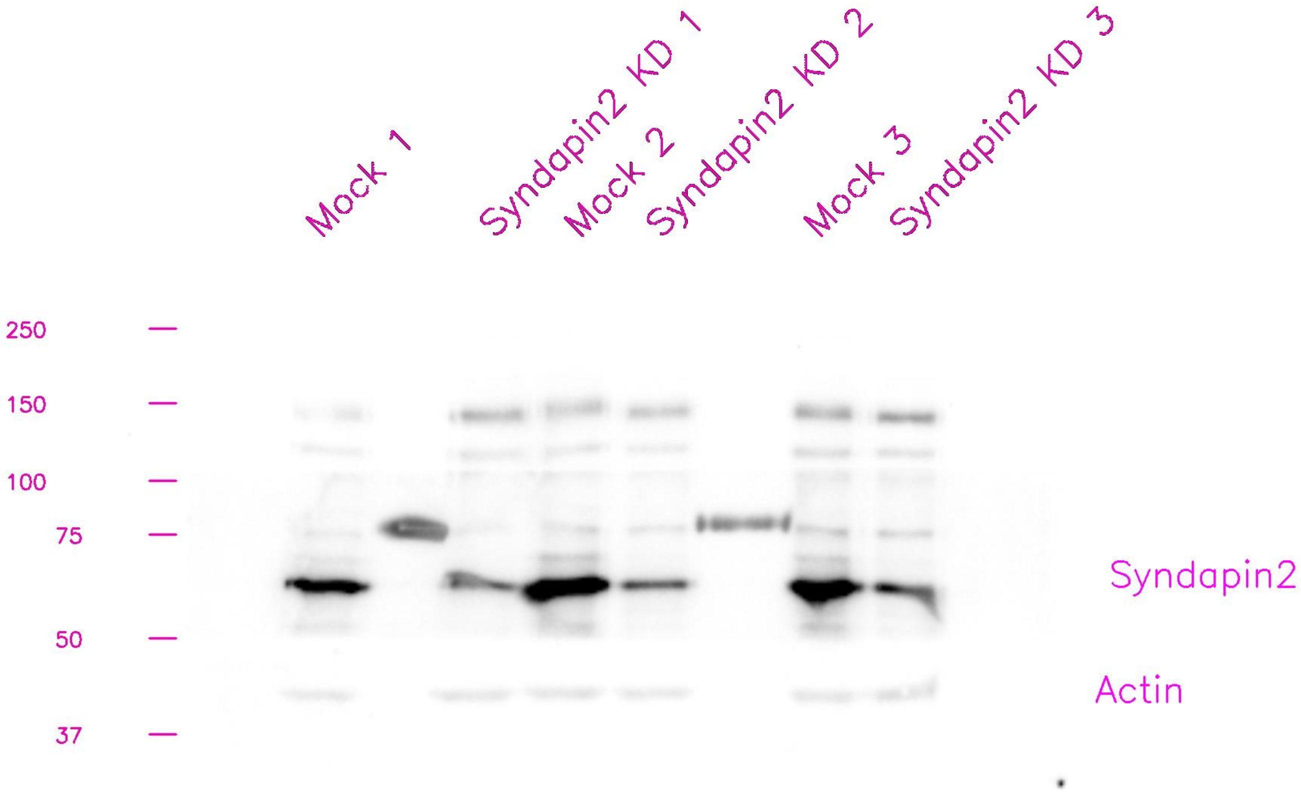
Mock 1
Syndapin2 KD 1
Mock 2
Syndapin2 KD 2
Mock 3
Syndapin2 KD 3
250
150
100
75
50
37
Syndapin2
Actin

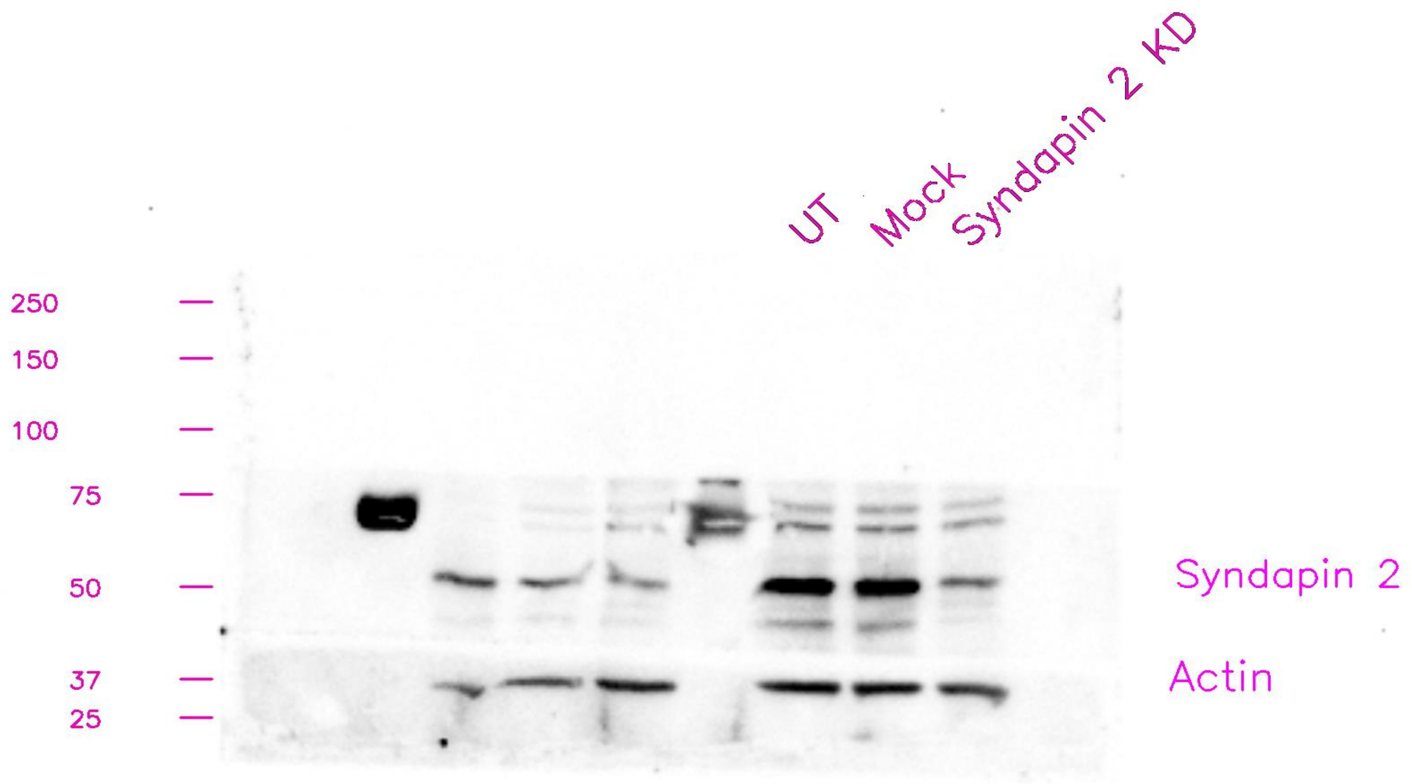
UT
Mock
Syndapin 2 KD
250
150
100
75
50
37
25
Syndapin 2
Actin

Fig. 8

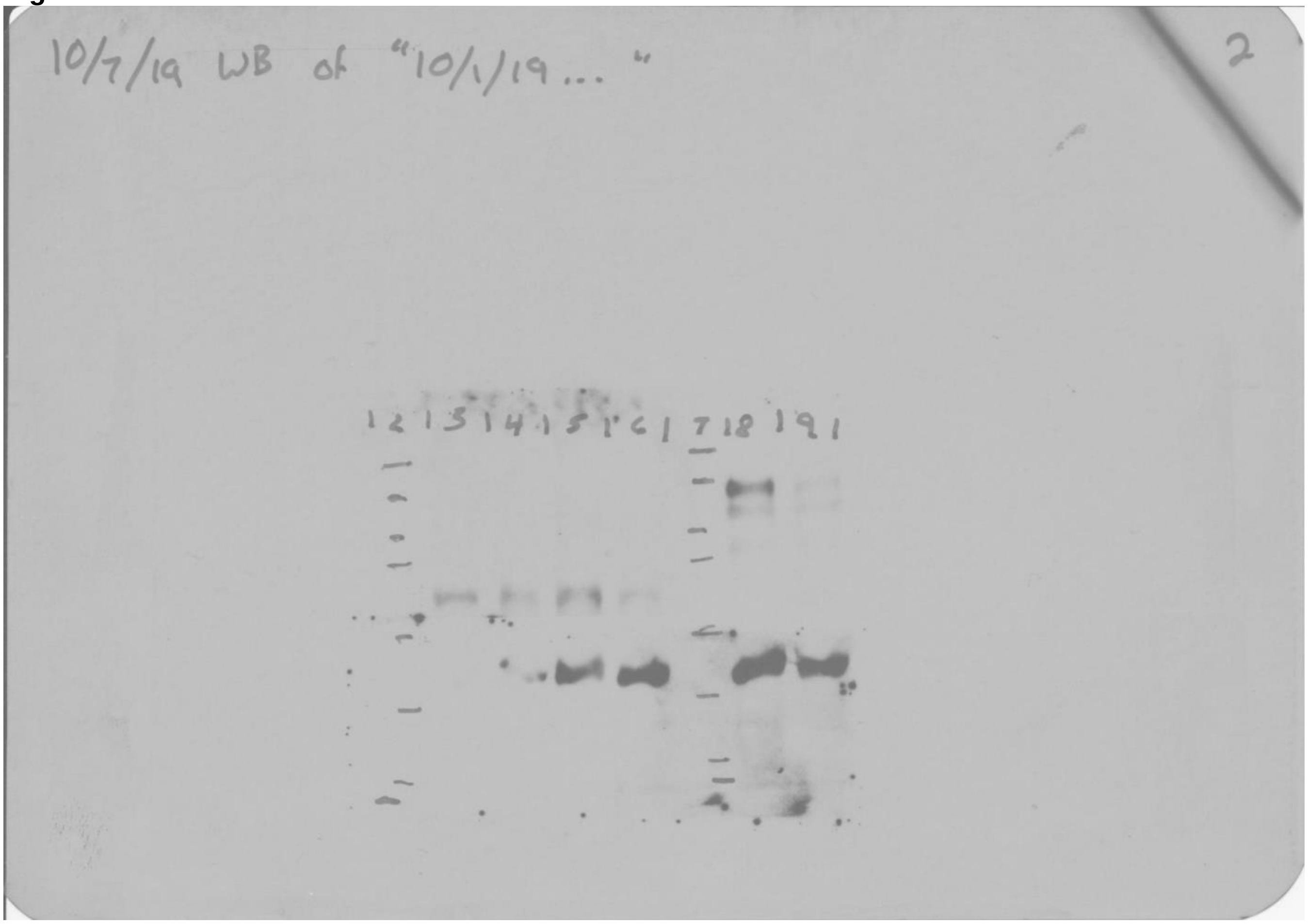
10/7/19 WB of "10/1/19..."
2

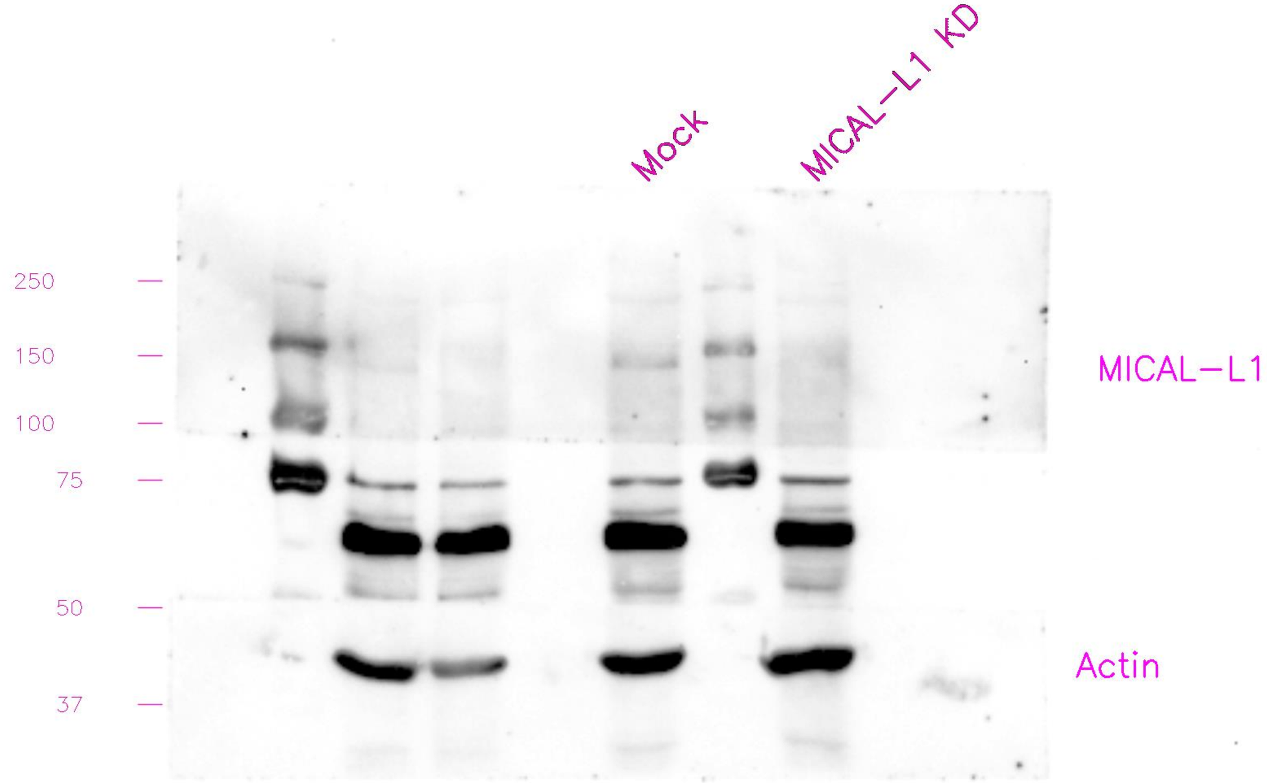
Mock
MICAL-L1 KD
250
150
100
75
50
37
MICAL-L1
Actin

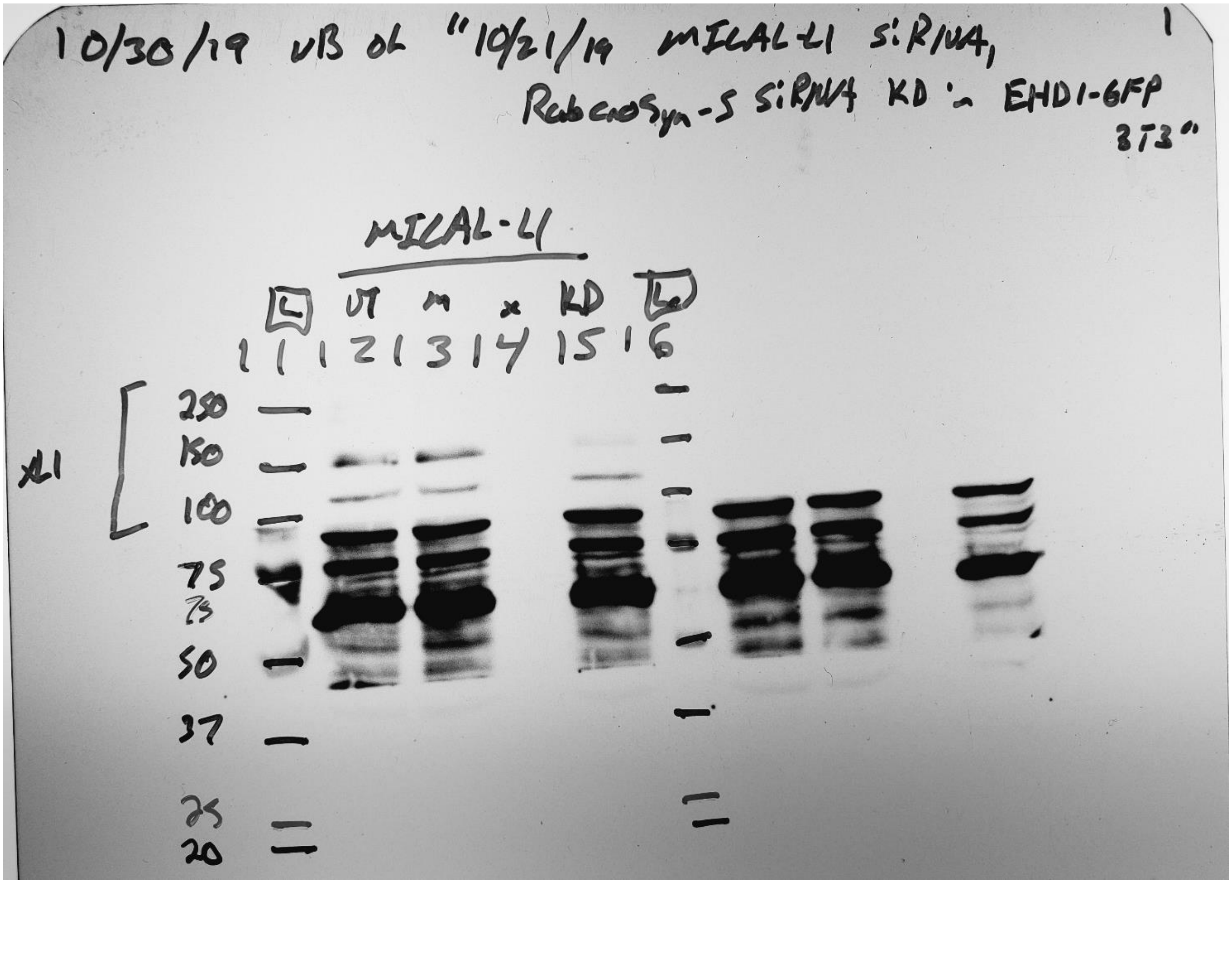
10/30/19 WB of "10/21/19 MICAL-L1 siRNA,
RabconSyn-5 siRNA KD in EHD1-GFP
3T3"
1
MICAL-L1
L
UT
M
x
KD
L
1
2
3
4
5
6
250
150
100
75
50
37
25
20
xL1

Sup. Fig. 1

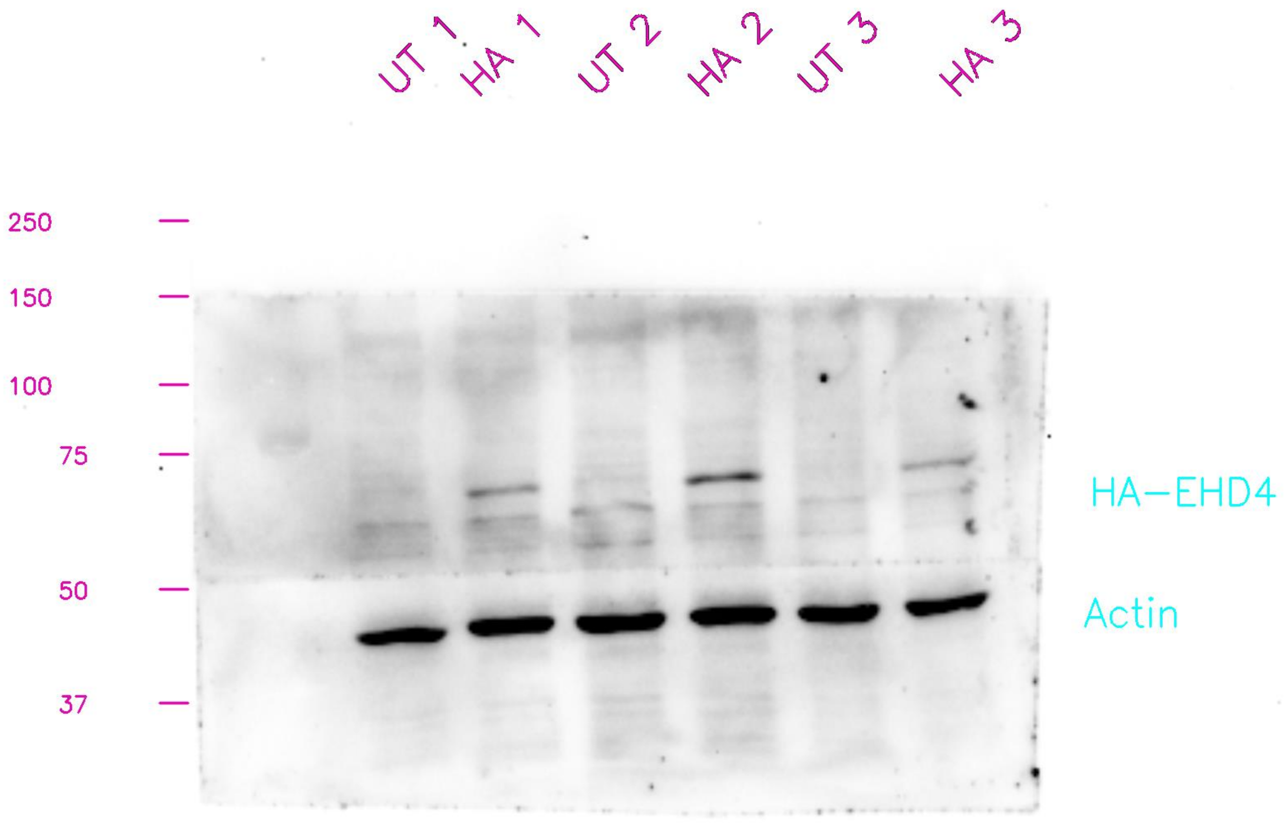

Sup. Fig. 2

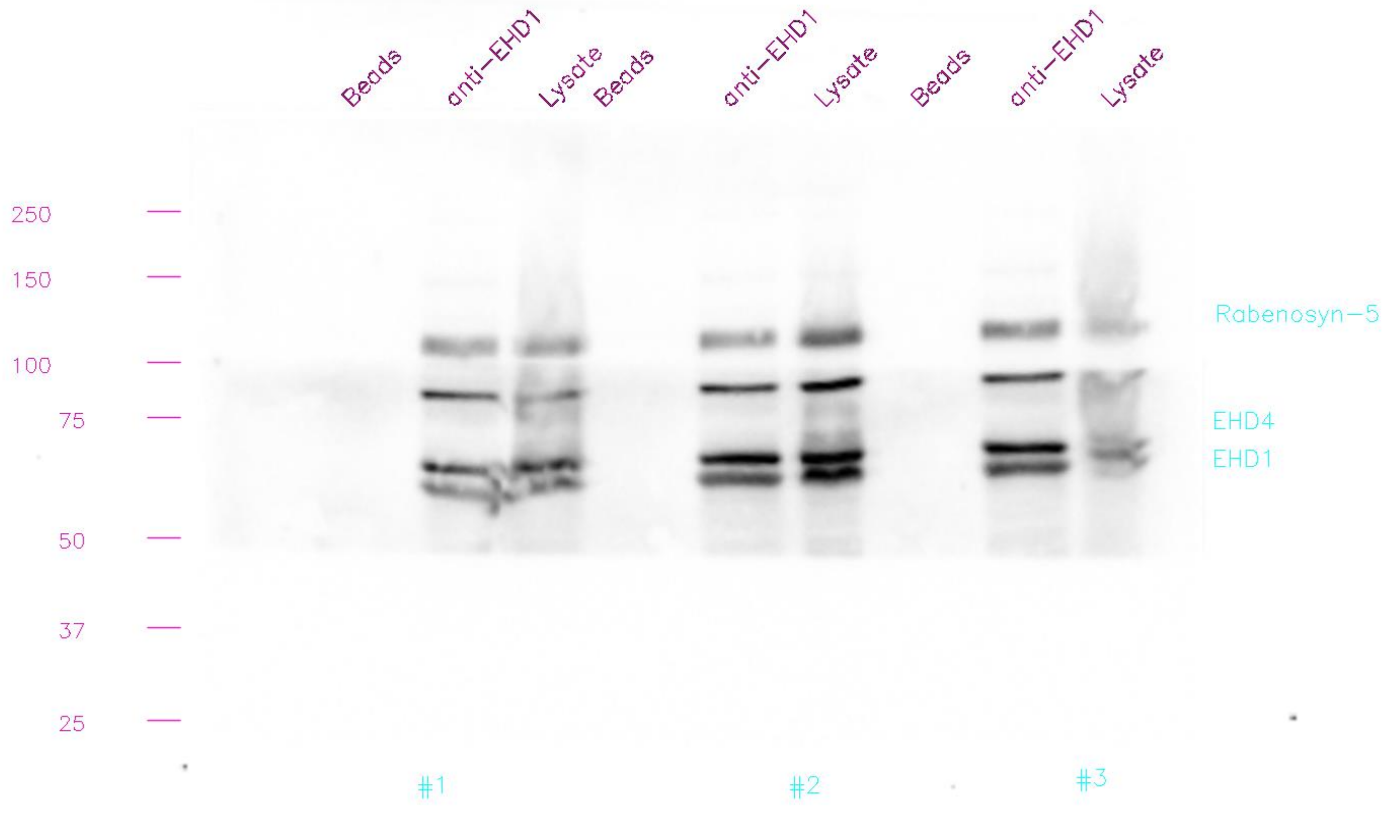

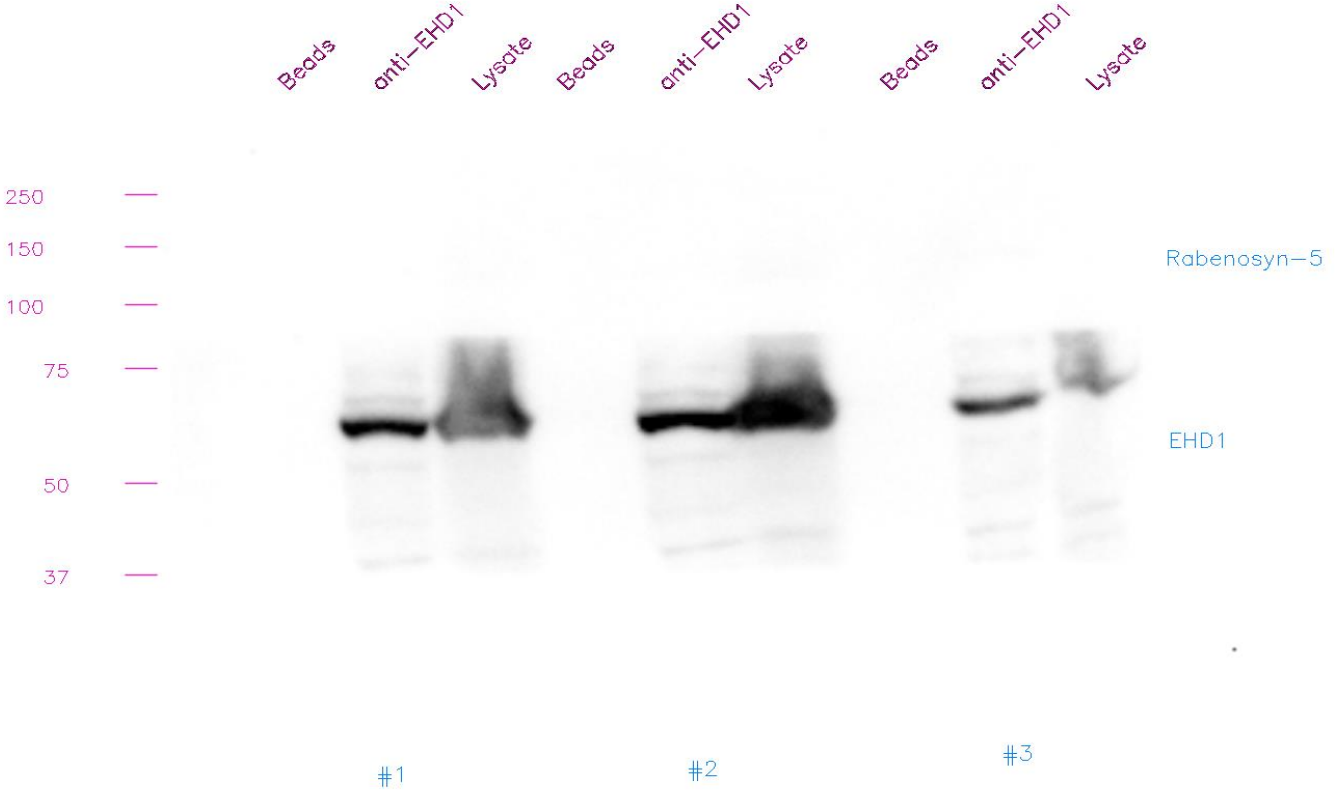
Beads
anti-EHD1
Lysate
Beads
anti-EHD1
Lysate
Beads
anti-EHD1
Lysate
250
150
100
75
50
37
Rabenosyn-5
EHD1
#1
#2
#3

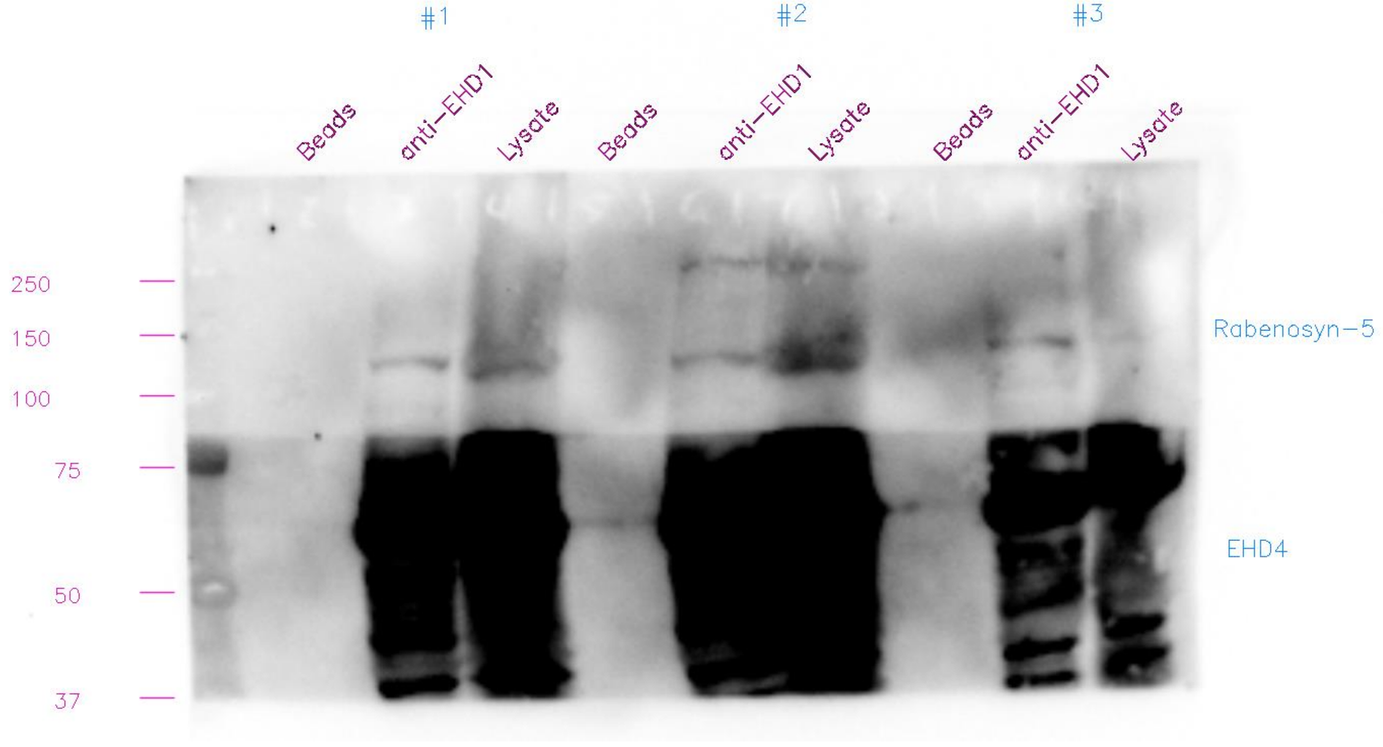
#1
#2
#3
Beads
anti-EHD1
Lysate
Beads
anti-EHD1
Lysate
Beads
anti-EHD1
Lysate
250
150
100
75
50
37
Rabenosyn-5
EHD4
